# Isolated splenic mucormycosis secondary to diabetic ketoacidosis: a case report

**DOI:** 10.1186/s12879-022-07564-3

**Published:** 2022-07-07

**Authors:** Shuai Luo, Xiang Huang, Yao Li, Jinjing Wang

**Affiliations:** Department of Pathology, Affiliated Hospital of Zunyi Medical University, Zunyi, Guizhou People’s Republic of China

**Keywords:** Mucormycosis, Splenic infarction, Diabetic ketoacidosis, Amphotericin B

## Abstract

**Background:**

Mucormycosis is a rare but serious opportunistic fungal infection that occurs in immunocompromised individuals, especially those with diabetic ketoacidosis. Presently, early diagnosis of the disease remains a challenge for clinicians.

**Case presentation:**

The patient, a 68-year-old woman with type 2 diabetes mellitus, was admitted with paroxic sharp pain in the left upper abdomen. CT imaging revealed a patchy hypodense shadow of the spleen with wedge-shaped changes. The patient was not considered early for fungal infection. The diagnosis of spleen mucormycosis was not confirmed until pathological biopsy after splenectomy. After surgery, blood glucose level was controlled, acidosis was corrected, and antifungal therapy was effective.

**Conclusions:**

We report here, for the first time ever, a case of isolated splenic mucormycosis secondary to diabetic ketoacidosis that was diagnosed and treated with antifungal drugs and splenectomy. Following splenectomy, the presence of splenic mucormycosis was confirmed when characteristic mycelia were observed in a tissue biopsy. As the location of any fungal infection is extremely relevant for treatment options and prognoses, early diagnosis and clinical intervention can greatly affect outcomes and prognoses for patients.

## Background

Mucormycosis is a rare invasive fungal infection that occurs mainly as a secondary condition in individuals with other underlying diseases (particularly diabetic ketoacidosis) that cause immune deficiency [1]. Based on the infected organ and clinical presentation, mucormycosis is usually divided into six clinical forms, namely, rhinocerebral, pulmonary, cutaneous, gastrointestinal, disseminated, and miscellaneous. Rhinocerebral mucormycosis is the most common form that occurs in diabetes patients with ketoacidosis; this form of mucormycosis rarely progresses to pulmonary or disseminated forms [2]. Mucormycosis is extremely rare, and isolated cases occurring in the spleen are rarer. Since the disease is extremely aggressive with a high mortality rate, early diagnosis and treatment are crucial for patient survival. This article provides some interesting points, which may be of value for the future clinical management of mucormycosis.

## Case presentation

**Fig. 1:**
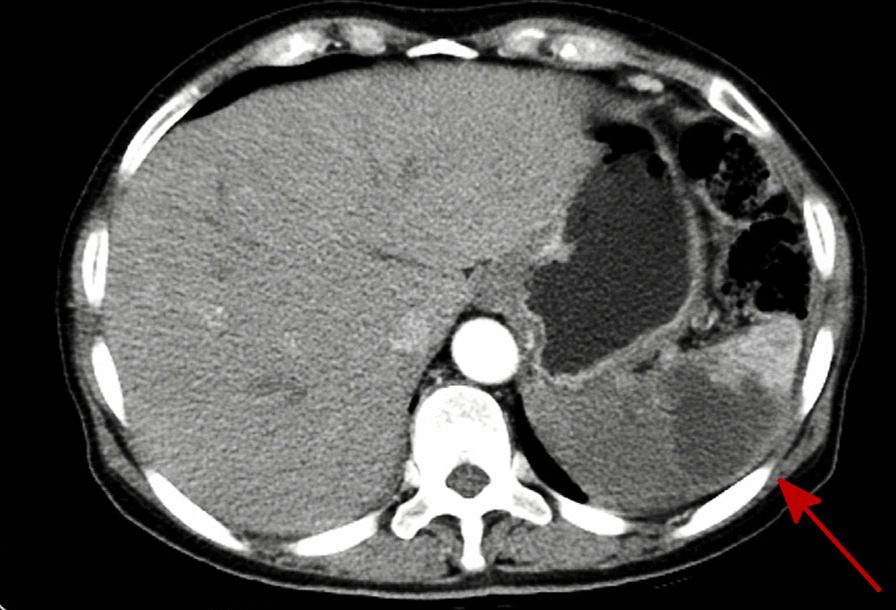
Abdominal computed tomography (CT) imaging revealed patchy hypodense shadow of the spleen with wedge-shaped changes

A 68-year-old woman with type 2 diabetes mellitus with poor glycaemic control was admitted with paroxic sharp pain in the left upper abdomen. She had a history of diabetes for 8 years and took metformin orally to control blood glucose; her blood glucose levels were stable. However, in the past six months, the fasting blood glucose was between 8 and 10 mmol/L, and the blood glucose at 2 h postprandial was between 12 and 18 mmol/L. There was no history of autoimmune diseases or long-term use of immunosuppressive drugs such as glucocorticoids. Physical examination showed tenderness in left upper abdomen, flat and soft abdomen, no gastrointestinal type and peristaltic wave, no varicose veins in the abdominal wall, no rebound tenderness, and no palpable liver and spleen. Laboratory analyses revealed that her blood glucose level was 32.83 mmol/L, C-reactive protein was 45.683 mg/L, HbA1c was 14.43%, urinary ketone bodies (++), and coagulation function tests reported the following: fibrinogen level 6.36 g/L, activated partial thromboplastin time 22.50 s, D-dimer level 2.28 mg/L, and fibrin degradation products 6.10 mg/L. Arterial blood gas analysis revealed a state of metabolic acidosis: pH 6.80, partial pressure of carbon dioxide in arterial blood (PaCO_2_) < 10 mmHg, bicarbonate (HCO_3_) < 10 mmol/L, and base excess − 33.8. Laboratory findings suggested hyperglycaemia with glycosuria and ketoacidosis. An abdominal computed tomography (CT) imaging revealed a patchy hypodense shadow of the spleen with wedge-shaped changes (Fig. 1). After admission, the patient was diagnosed as having diabetic ketoacidosis combined with spleen infarction, which was life-threatening, based on clinical characteristics and auxiliary examination.

**Fig. 2:**
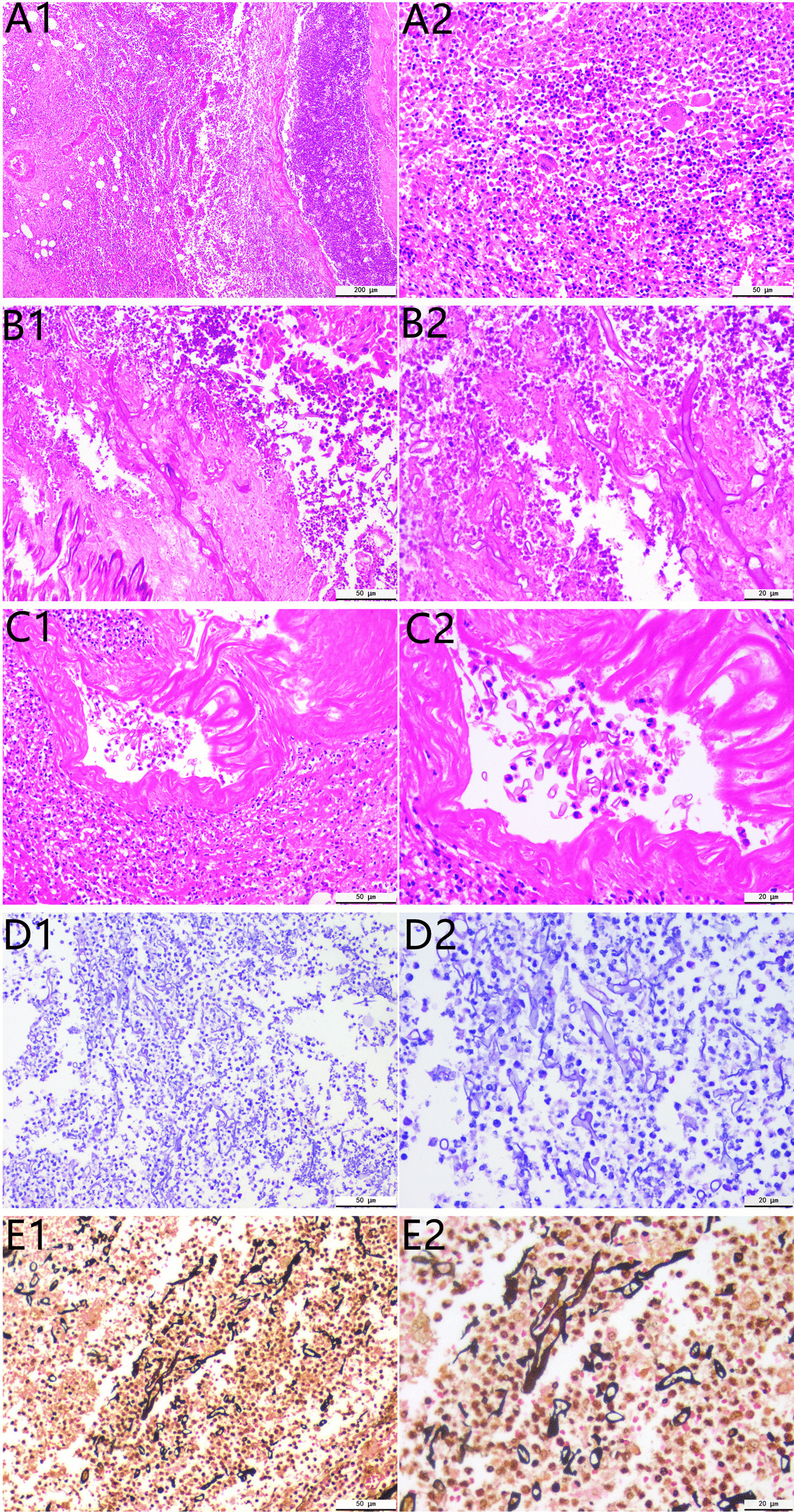
Histopathological examination shows a large amount of coagulated necrotic tissue containing neutrophils, lymphocytes, eosinophils, multinucleated giant cells, and necrotic tissue (**A1** H&E stain ×50; **A2** H&E stain ×200). Folded, wrinkled, or swollen broad tubular hyphae are clearly observed under high magnification. Disordered hyphae rarely show branching; a few are visible as right-angled branches detached from the lateral wall, and the rest are seen as transversely cut round, oval, and polygonal unbranched cystic hyphae (**B1** H&E stain ×200; **B2** H&E stain ×400). Massive mycelial invasion is seen in the necrotic area of the spleen and in the blood vessels, with neutrophil infiltration both inside and outside the vessels, leading to fungal vasculitis (**C1** H&E stain ×200; **C2** H&E stain ×400). Periodic acid Schiff (PAS) stain shows the mycelium with pink walls (**D1** PAS stain ×200; **D2** PAS stain ×400). Grocott methylamine silver (GMS) stain shows the mycelium with brown–black walls (**E1** GMS stain ×200; **E2** GMS stain ×400)

Therefore, emergency splenectomy was performed, and routine spleen histopathology showed a large amount of coagulated necrotic tissue containing neutrophils, lymphocytes, eosinophils, multinucleated giant cells, and necrotic tissue (Fig. 2A1,2). Broadly tubular hyphae that were folded, wrinkled, or swollen (10–15 μm) were clearly observed under high magnification. The disordered hyphae rarely branched, though a few were seen to branch at right angles separating from the lateral wall. Transversely cut, round, elliptical, or polygonal unbranched cystic hyphae were seen in other parts of the tissue (Fig. 2B1,2). Massive mycelial invasion was seen in the necrotic areas of the spleen and in the blood vessels, where mycelial and neutrophil infiltration leading to fungal vasculitis was also observed (Fig. 2C1,2). Grocott methylamine-silver (GMS) (Fig. 2D1,2) and periodate-Schiff (PAS) (Fig. 2E1,2) were used to stain the fungal walls purplish-red and brownish-black, respectively, and the results clearly revealed structural details of the walls of the fungal hyphae.

After surgery with rehydration correct dehydration, acidosis and electrolyte disorder, antibiotics to prevent infection, intravenous antifungal therapy (one 2-week course of amphotericin B in liposome form, approx. 5 mg/kg). After 12 h of intravenous infusion of insulin (blood glucose was measured every hour), levels of blood ketone body and urine ketone body turned negative for three consecutive times, the intravenous infusion of insulin was stopped, and the blood glucose was controlled by insulin pump, with close monitoring via blood gas analysis until the acid-base balance was achieved.

After discharge, metformin was prescribed regularly to control blood glucose. The patient was followed up for 2 years without relapse.

## Discussion

Mucormycosis is a life-threatening and angio-invasive fungal infection that often occurs secondary to diabetic ketoacidosis. Thus far, no specific clinical symptoms and ancillary tests have been identified for this condition, which makes the clinical diagnosis of mucormycosis very difficult.

The occurrence of isolated splenic mucormycosis is extremely rare, as till now, only five cases of this condition have been reported [3–7] (Table 1). The underlying conditions in these cases were human immunodeficiency virus infection, multiple myeloma, aplastic anaemia, acute lymphoblastic leukaemia, and lung transplant.

**Table 1:** Clinical characteristics and management of isolated splenic mucormycosis reported in the literature

References		Age (year)	Sex	Risk factor	Revealing symptoms	Splenic CT scan findings	Therapy	Outcome
Teira et al. [3]	1993	33	M	HIV	Fever, mild epigastric discomfort	2 areas of low density in the spleen	AMPH-B,splenectomy	Alive
Pastor et al. [4]	1999	64	M	Multiple myeloma, chemotherapy, adenocarcinoma in the left colon	Rectal hemorrhage	Hypodense mass in the spleen	L-AMB, Segmentary, colectomy, splenectomy	Dead
Nevitt et al. [5]	2001	16	F	Acute lymphoblastic leukemia, chemotherapy	Left upper quadrant pain	Multiple hypodense lesions in the spleen	AMPH-B,splenectomy	Alive
Connor et al. [6]	2018	46	F	Pulmonary fibrosis, double lung ransplant, IS	Haematemesis, malaena	NS	L-AMB + PCZ switched to PCZ, Splenectomy, gastrostomy, duodenostomy	Alive
Sharma et al. [7]	2018	14	M	None	Fever,left upper abdominal pain, nausea, vomiting	Completely infarcted spleen	AMPH-B, splenectomy	Dead
Our case	2022	68	F	Diabetic ketoacidosis	Pain in the left upper abdomen	A patchy hypodense shadow of the spleen with wedge-shaped changes	AMPH-B, splenectomy	Alive

As of now, no cases of isolated splenic mucormycosis secondary to diabetic ketoacidosis have been reported. In Roden’s review of 929 cases of mucormycosis [8], diabetes was the primary risk factor (36%), with the most common sites of infection being the nasal sinuses, orbit, and brain. Patients with diabetes have low immune function, and the phagocytosis and chemotaxis abilities of neutrophils and macrophages are limited, enabling fungal germination and mycelium growth. Iron reuptake by transferrin and ferritin is also reduced, which will increase free iron and stimulate rapid mucosal proliferation [9]. Usually, mucormycosis spores are known to enter the body through respiratory inhalation, gastrointestinal ingestion, contaminated wounds caused by skin loss, intravenous infusion, etc. [2, 10]. The route of infection in isolated splenic mucormycosis is controversial with some suggesting that it may be blood-borne [11]; however, the specific underlying mechanism has not been clearly identified.

The clinical manifestations of splenic mucormycosis are nonspecific [12] and may present as pain in the upper left part of the abdomen, occasionally radiating to the left shoulder, and may be accompanied by common symptoms of infection, such as fever. Other symptoms may include gastrointestinal symptoms such as nausea and vomiting, with pressure-induced pain in the upper left part of the abdomen on examination. Low-density images of the spleen, which may be patchy or wedge-shaped, are also commonly seen in abdominal CTs, suggesting splenic infarction [13].

As of now, fungal cultures, histopathological biopsies and molecular biology-based tools may be used to confirm a diagnosis for mucormycosis. The fungal culture is highly specific, but low sensitivity results in false negatives in more than half the cases of mucormycosis infections. Histopathological biopsies can identify fungal infections more clearly, while also allowing visualisation of vascular invasion by the fungus [14].

An important hallmark of mucormycosis is vascular invasion and extensive tissue infarction, with the infected organ/area often showing intense granulomatous inflammation (with epithelioid cells, multinucleated giant cells, lymphocytes, and necrotic tissue) [15]. The infected areas also show massive neutrophil and eosinophil infiltration, interstitial fibrosis, and granulation tissue formation, sometimes with the classic Splendore-Hoeppli phenomenon [16].

The fungal hyphae are wide (5–20 μm), thin-walled, and irregularly ribboned with little separation (pauciseptate), and branch irregularly at no fixed angle; in cross-sections, the fungus appears cystic, and spores are rarely seen [15]. Haematoxylin and eosin staining of the mycelia reveals only the cell wall with no internal structures; however, GMS and PAS, which stain the fungal cell walls purplish-red and brownish-black, respectively, are better at revealing fungal wall structures [15]. New molecular biology-based methods can also confirm the presence of mucormycosis but are not yet widely used in clinical practice; more advanced non-invasive tests for this condition have yet to be developed [15].

In this case, the lungs, nose, brain, or skin were found to be free of mucormycosis. Since the spleen is an intra-abdominal parenchymal organ, the diagnosis of mucormycosis splenicum was confirmed mainly through histopathological biopsy. Splenic mucormycosis is mainly diagnosed by comparing biopsies of infected tissue with other splenic tissue infected by other fungal agents such as Aspergillus and Candida; Candida hyphae are thin (2–4 μm in diameter) and often bead-like [18], whereas Aspergillus hyphae are moderately and uniformly thick (5–10 μm in diameter) and usually exhibit acute angular branching [19].

In this case, the splenic mucormycosis was treated through surgical splenectomy and administration of an antifungal agent (amphotericin B), along with symptomatic treatment (acid correction, rehydration, etc.). However, some characteristics of the patients might have also influenced the treatment decision; for example, the patient in this case was older and had a history of diabetes for many years. During the treatment, a large amount of fluid rehydration is likely to induce acute left heart failure, which is life-threatening [20]. Since mucormycosis is associated with high mortality rates, early and definitive diagnosis and clinical intervention can directly affect prognosis [13].

In conclusion, we report here, the first known case of isolated splenic mucormycosis occurring secondary to diabetic ketoacidosis. The diagnosis was confirmed by microscopy of the splenic biopsy tissue, in which characteristic mycelia were observed. The patient was treated with an antifungal agent (amphotericin B) after surgically removing the spleen, and significant improvement was seen. This case serves as an important reference for the early diagnosis and treatment of isolated splenic mucormycosis associated with diabetic ketoacidosis.

## Data Availability

All the data regarding the findings are available within the manuscript.

## Abbreviations

CT: Computed tomography
PAS: Periodicacid–Schiff
GMS: Gomori methenamine silver
